# RRM1 single nucleotide polymorphism -37C→A correlates with progression-free survival in NSCLC patients after gemcitabine-based chemotherapy

**DOI:** 10.1186/1756-8722-3-10

**Published:** 2010-03-13

**Authors:** Song Dong, Ai-Lin Guo, Zhi-Hong Chen, Zhen Wang, Xu-Chao Zhang, Ying Huang, Zhi Xie, Hong-Hong Yan, Hua Cheng, Yi-Long Wu

**Affiliations:** 1 Guangdong Lung Cancer Institute, Guangdong General Hospital, Guangdong Academy of Medical Sciences, Guangzhou 510080, China; 2 Southern Medical University, Guangzhou 510515, PR China; 3 Thoracic Surgery Department, the Fifth Affiliated Hospital of Sun Yet-sen University, Zhuhai 519000, China

## Abstract

**Background:**

The ribonucleotide reductase M1 (RRM1) gene encodes the regulatory subunit of ribonucleotide reductase, the molecular target of gemcitabine. The overexpression of RRM1 mRNA in tumor tissues is reported to be associated with gemcitabine resistance. Thus, single nucleotide polymorphisms (SNPs) of the RRM1 gene are potential biomarkers of the response to gemcitabine chemotherapy. We investigated whether RRM1 expression in peripheral blood mononuclear cells (PBMCs) or SNPs were associated with clinical outcome after gemcitabine-based chemotherapy in advanced non-small cell lung cancer (NSCLC) patients.

**Methods:**

PBMC samples were obtained from 62 stage IIIB and IV patients treated with gemcitabine-based chemotherapy. RRM1 mRNA expression levels were assessed by real-time PCR. Three RRM1 SNPs, -37C→A, 2455A→G and 2464G→A, were assessed by direct sequencing.

**Results:**

RRM1 expression was detectable in 57 PBMC samples, and SNPs were sequenced in 56 samples. The overall response rate to gemcitabine was 18%; there was no significant association between RRM1 mRNA expression and response rate (*P *= 0.560). The median progression-free survival (PFS) was 23.3 weeks in the lower expression group and 26.9 weeks in the higher expression group (*P *= 0.659). For the -37C→A polymorphism, the median PFS was 30.7 weeks in the C(-)37A group, 24.7 weeks in the A(-)37A group, and 23.3 weeks in the C(-)37C group (*P *= 0.043). No significant difference in PFS was observed for the SNP 2455A→G or 2464G→A.

**Conclusions:**

The RRM1 polymorphism -37C→A correlated with PFS in NSCLC patients treated with gemcitabine-based chemotherapy. No significant correlation was found between PBMC RRM1 mRNA expression and the efficacy of gemcitabine.

## Background

Lung cancer is a leading cause of cancer deaths in both China and the USA [1,2]. More than 75% of lung cancers are non-small cell lung cancer (NSCLC) [3]. Most patients have advanced NSCLC when diagnosed, and chemotherapy is one of the major treatment options in these patients. A meta-analysis showed the importance of gemcitabine in the treatment of advanced NSCLC; median survival with gemcitabine-based chemotherapy was 9 months, versus 8.2 months with non-gemcitabine combinations [4]. However, resistance to gemcitabine or relapse soon after treatment has limited the efficacy of this drug.

The molecular target of gemcitabine is ribonucleotide reductase [5]. This enzyme catalyzes the rate-limiting step in deoxyribonucleotide formation and is the only known enzyme that converts ribonucleotides to deoxyribonucletides, which are required for DNA polymerization and repair [6]. The RRM1 gene encodes the regulatory subunit of ribonucleotide reductase; diphosphorylated gemcitabine (dFdDDP) indirectly inhibits DNA synthesis through the inhibition of RRM1 [7].

In patients with advanced NSCLC, RRM1 mRNA expression levels are related to the efficacy of gemcitabine therapy. Retrospective studies of stage IV NSCLC patients treated with gemcitabine-based chemotherapy have shown that patients with low tumor RRM1 mRNA levels lived longer than patients with higher expression levels [8-11]. Furthermore, the efficacy of gemcitabine plus docetaxel can be improved when specifically administered according to the tumor mRNA expression of BRCA1, RRM1, and RRM2. An association between RRM1 overexpression and resistance to gemcitabine has been observed in the laboratory [12,13]. Thus, customized chemotherapy based on tumor RRM1 expression is a reasonable strategy for advanced NSCLC patients. Nevertheless, it is difficult to ordinarily use tumor RRM1 mRNA levels as a predicator to determine optimal chemotherapy regimens in clinical practice. As some advanced NSCLC patients are diagnosed only by cytopathology or needle biopsy with a small amount of tumor tissue, insufficient material may be available for gene expression analysis. More convenient and precise biomarkers are needed.

SNPs represent natural genetic variability at a high density in the human genome and have been confirmed as predictive markers of some treatment responses [14]. An advantage of SNPs as predictive markers is that genomic DNA can be analyzed from samples of PBMCs, even when tumor mRNA is not available from patients with advanced NSCLC. An adenine→cytosine substitution in the 5' non-coding region of RRM1, located 37 nucleotides upstream of the start codon, has been associated with higher RRM1 expression levels[15]. Furthermore, -37C→A alone and the allelotypes C(-)37A-C(-)524T were related to chemotherapy outcome in clinical trials[16,17].

In this study, we examined RRM1 mRNA expression in PBMCs by real-time reverse transcription PCR and analyzed the SNPs by direct sequencing. The possibility of using PBMC RRM1 expression or SNPs as efficacy predictors in NSCLC patients treated with gemcitabine was tested.

## Results

### Patient characteristics and efficacy of treatment

Between March 2006 and February 2007, 62 eligible patients were enrolled. The patients' ages ranged from 35 to 70 years (median, 61); 21 were women. Among the 62 patients, 59 were naive to any previous anticancer treatment, two had suffered recurrences after surgical resection, and one had received whole-brain radiotherapy. All patients received at least one cycle of chemotherapy. Baseline characteristics of the 62 patients are shown in Table 1. No patient had CR, 11 patients had PR, 44 patients had SD, and 7 patients had PD. The median progression-free survival (PFS) was 22.8 weeks.

**Table 1:** Baseline characteristics of the 62 patients

Characteristic	*n *(%)
Gender	
Male	40 (64.5)
Female	22 (35.5)
Age	
≤ 65 years	50 (80.6)
>65 years	12 (19.4)
WHO PS	
0	11 (17.7)
1	51 (82.3)
Histology	
Squamous cell carcimoma	11 (17.7)
Adenocarcinoma	46 (74.2)
Large cell carcinoma	3 (4.8)
Other NSCLC	2 (3.3)
Stage	
IIIA	2 (3.3)
IIIB	10 (16.1)
IV	50 (80.6)
Weight loss ≥5%	
Yes	13 (21.0)
No	47 (75.8)
Unknown	2 (3.2)

### RRM1 expression and treatment efficacy

Amplification of RRM1 was successful in 57 samples, and we failed to extract RNA from five blood samples. There was considerable variation in the expression level, with relative expression values ranging from 1.81 × 10^-6 ^to 7.78 × 10^-2 ^(median, 1.54 × 10^-4^; mean, 6.48 × 10^-3^). Patients were divided into two groups, those with expression equal to or higher than the median and those with expression below the median. No differences in clinical characteristics, including age, gender, histological type, and stage, were observed between the groups, and there was no significant association between RRM1 expression and response (*P *= 0.560). Table 2 shows the baseline characteristics and response according to RRM1 expression in PBMCs.

**Table 2:** Baseline characteristics by RRM1 expression

Characteristic	RRM1 mRNA Expression^1^		*P *value
		
	Low	High	
Age, years			
≥ 62	14	11	
<62	14	18	0.592
Gender			
Male	20	19	
Female	8	10	0.501
WHO PS			
0	2	9	
1	26	20	0.062
Smoking			
Yes	15	13	
No	13	16	0.207
Weight loss ≥ 5%			
Yes	4	8	
No	24	19	
Unknown^2^	0	2	0.348
Histology			
Adenocarcinoma	19	22	
Squamous cell			
carcinoma	6	6	
Others^2^	3	1	0.833
Stage			
III	3	7	
IV	25	22	0.257

1 mRNA from 57 samples was available for the analysis of RRM1 expression.

2 These groups were excluded from the statistical analysis.

**Figure 1:**
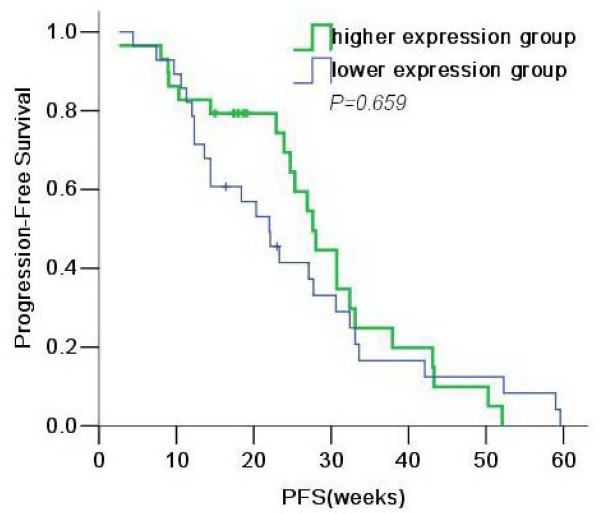
**Kaplan-Meier survival estimates for patients with NSCLC, based on RRM1 mRNA expression in PBMCs**.

We used a log-rank test to analyze the level of significance between PFS and RRM1 expression. The median PFS was 23.3 weeks (95% CI, 15.3-31.3) in the lower-expression group and 23.9 weeks (95% CI, 22.8-31.0) in the higher-expression group, with no significant association between RRM1 mRNA expression and PFS (*P *= 0.659; Fig. 1).

### SNP genotype and efficacy of gemcitabine

**Figure 2:**
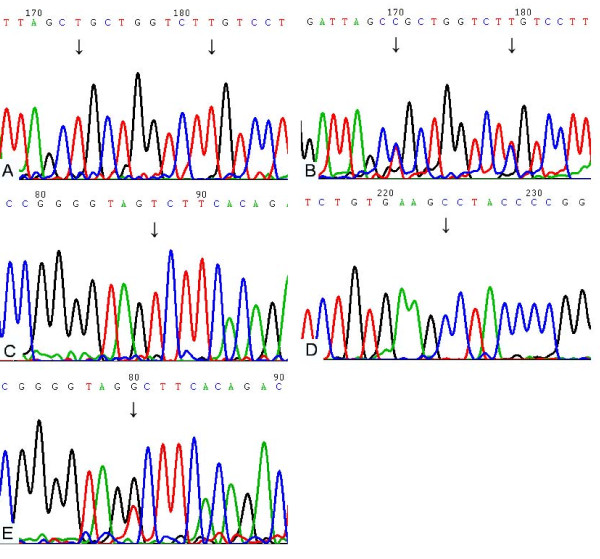
**Sequence chromatograms for polymorphisms are shown**. The arrows indicate the polymorphic positions. A: A2455A and A2464A (antisense). B: A2455G and G2464A (antisense). C: A(-)37A (antisense). D: C(-)37C (sense). E: A(-)37C (antisense).

**Figure 3:**
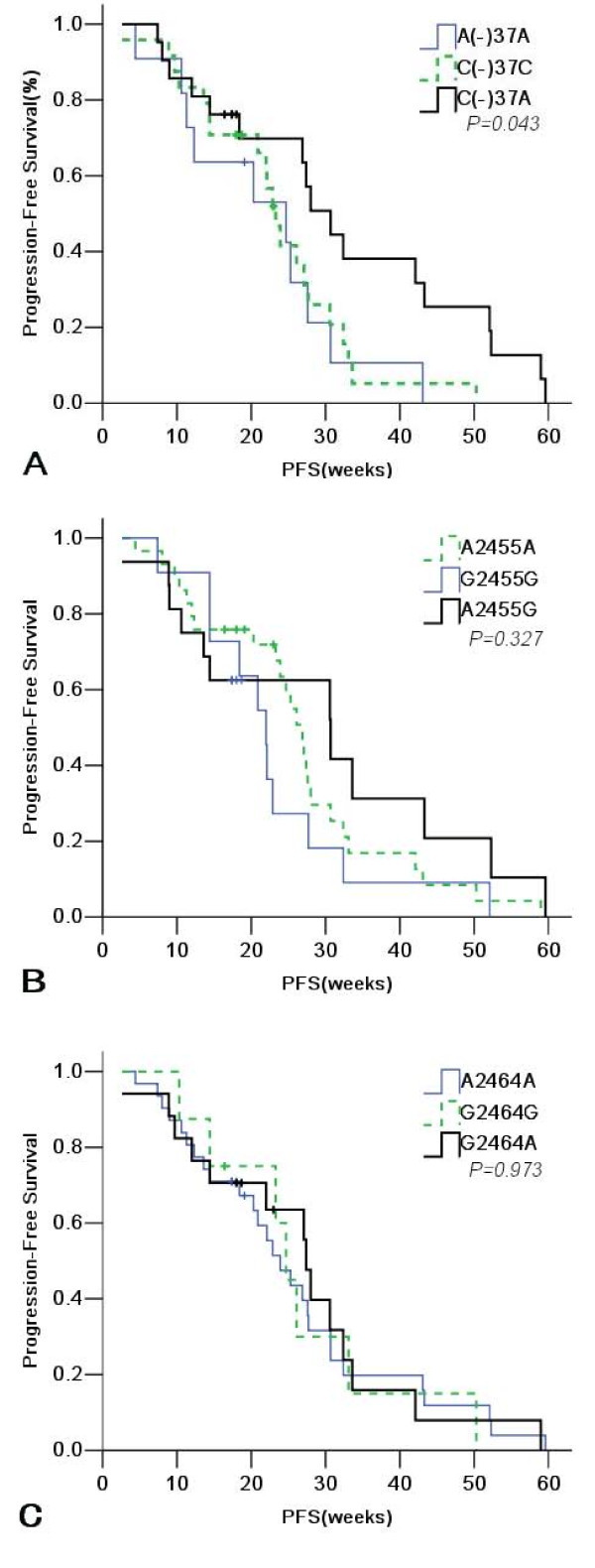
**Kaplan-Meier survival estimates based on RRM1 SNPs (A-C)for patients with NSCLC**.

Blood samples from 56 patients were available for the analysis of RRM1 SNPs. An analysis of sequence chromatograms revealed RRM1 polymorphisms (Fig. 2). The allele frequencies for -37C→A were 0.196 (11/56) for A(-)37A, 0.428 (24/56) for C(-)37C, and 0.376 (21/56) for C(-)37A; for 2455A→G, 0.482 (27/56) for A2455A, 0.214 (12/56) for G2455G, and 0.304 (17/56) for A2455G; and for 2464G→A, 0.554 (31/56) for A2464A, 0.142 (8/56) for G2464G, and 0.304 (17/56) for G2464A. Kendall's tau correlation was used to test the relationship between genotype and chemotherapy response, but no significant association was found (-37C→A, *P *= 0.514; 2455A→G, *P *= 0.849; 2464G→A, *P *= 0.191). For the polymorphism -37C→A, the median PFS was 30.7 weeks (95% CI, 24.5-36.9) for the C(-)37A genotype, 24.7 weeks (95% CI, 6.8-42.6) for A(-)37A, and 23.3 weeks for C(-)37C (95% CI, 20.8-25.8; *P *= 0.043). No genotype of 2455A→G or 2464G→A showed a significant correlation with sensitivity to gemcitabine (Table 3; Fig. 3A-C).

**Table 3:** Response and PFS by RRM1 SNPs

RRM1 SNPs	Response^1 ^(*n*)			*P *value^2^	PFS(weeks)	*P *value^3^
				
	PR	SD	PD			
A(-)37A	1	7	2		24.7	
C(-)37C	2	20	2		23.3	
A(-)37C	6	14	2	0.514	30.7	0.043
A2455A	5	22	2		26.9	
G2455G	2	6	3		22.0	
A2455G	2	13	1	0.849	30.7	0.327
G2464G	0	7	1		23.9	
A2464A	6	20	5		24.7	
G2464A	3	13	1	0.191	27.4	0.973

1 Genomic DNA from 56 patients was available for the analysis of RRM1 SNPs and response.

2 Kendall's tau correlation

3 Kaplan-Meier survival estimates.

Abbreviations: PR, Partial response; SD, Stable disease; PD, Progressive disease; PFS, Progression-free survival.

### RRM1 genotype and mRNA expression

Paired DNA/mRNA was successfully extracted from 53 blood samples. The mRNA expression levels were compared according to SNP genotype, and no significant difference was found (-37C→A, *P *= 0.693; 2455A→G, *P *= 0.081; 2464G→A, *P *= 0.650).

### RRM1 genotype and toxicity

All patients who received at least one cycle of chemotherapy were included in the toxicity analysis. Hematological toxicity grade ≥ 2 was observed in 22 patients, and grade 3/4 was seen in 12 patients. Hepatotoxicity grade ≥ 2 was observed in two patients; vomiting grade ≥ 2, in two patients; and rash grade ≥ 2, in one patient. Hematological toxicity grade 3/4 was observed in 50% of patients (9/18) harboring A2455G and in 7.7% of patients (3/39) harboring homozygous G2455G or A2455A (r = 0.482, *P *< 0.001). No other significant difference was observed according to SNP genotype.

## Discussion

The use of gene expression as a predictive marker for the efficacy of chemotherapy is an important area of translational research. We wanted to know whether RRM1 mRNA expression in PBMCs could serve as a substitute for predicting the efficacy of gemcitabine-based chemotherapy. To test this, venous blood was collected before chemotherapy and gene expression was analyzed, but no association was found between RRM1 mRNA expression in PBMCs and the efficacy of gemcitabine treatment. We also analyzed RRM1 expression in lung tumors and adjacent normal lung tissue from 17 patients who had undergone surgery and found no significant association between RRM1 expression in lung tumor cells and in normal lung tissue (data no shown). In this study, all of the 62 patients were diagnosed with advanced NSCLC, so tumor tissue or normal lung tissue was not available for the analysis of any correlation between RRM1 expression in PBMCs and in normal tissue.

Ribonucleotide reductase is involved in the proliferation and metabolism of cells; the proliferative characteristics of cancer cells are different from those of pulmonary epithelial cells and other cells in normal lung tissue. On the other hand, the PBMCs mainly contain lymphocytes and monocytes which are critical in the immune system with different proliferative activity. So we speculated that the simple comparison of mRNA expression between PBMCs and cancer cells is unavailable.

Genetic polymorphisms may affect protein structure, function, stability, or folding. The most common form of polymorphism in the human genome is a SNP, and some SNPs have been shown to correlate with drug sensitivity and toxicity. In a previous study, we found that the intron 1 (CA) repeat genetic polymorphisms of the epidermal growth factor receptor (EGFR) gene were correlated with EGFR protein expression and clinical response in NSCLC patients treated with EGFR tyrosine kinase inhibitor[18]. To find markers that could predict gemcitabine sensitivity, we analyzed the SNPs of RRM1, the target of gemcitabine. Based on previous reports, we selected the polymorphism sites -37C→A, 2455A→G, and 2464G→A as target SNPs. The RRM1 polymorphism C(-)37A affects promoter activity *in vitro*[19] but the use of a single genetic polymorphism, -37C→A, as predictor was uncertain[17,20]. Gemcitabine sensitivity has been associated with RRM1 A2464A *in vitro *[21], but no similar result has been observed in breast cancer patients [22]. As mentioned above, the values of these SNPs were different in previous studies and we considered it necessary to analyze these SNP sites.

The -37C→A polymorphism is located in the promoter region, upstream of the transcriptional start point. Given that promoter activity is one of the factors controlling RRM1 expression, we expect that polymorphism at -37C→A affects promoter activity. We noticed that 27.3% of the patients (6/22) showing a partial response harbored C(-)37A, but only 8.8% of the patients homozygous at -37C→A had a partial response; This SNP had no significant association with response rates (*P *= 0.353). Limited by the period of study, only 62 patients were enrolled, we expected that relationship between SNPs and response could be understood if there were enough cases, but the PFS of patients with A(-)37C was significantly different from that of patients with the other genotypes (*P *= 0.043). Heterozygous A2455G was present in 50% of patients (9/18) with grade 3/4 hematological toxicity(r = 0.482, *P *< 0.001); thus, we suggest that patients harboring A2455G may be more susceptible to gemcitabine, although no significant association was observed between A2455G and chemotherapy outcome, maybe this is due to the limitation of sample size. The SNPs 2455A→G and 2464G→A are located at the end of the RRM1 cDNA; as both are synonymous SNPs, the amino acid would not be different among the genotypes. However, a previous report showed that a synonymous SNP in RRM1 gene was correlated with gene expression level [23]. We hypothesize that the 2455A→G polymorphism may affect the efficiency of RRM1 mRNA transcription, resulting in different mRNA expression levels; this needs further investigation.

Based on our results, we cannot determine whether the RRM1 mRNA expression level in PBMCs is useful in predicting the efficacy of gemcitabine-based chemotherapy. However, regarding SNPs, patients harboring the C(-)37A genotype had a longer PFS with gemcitabine-based chemotherapy than patients with the other SNPs. Studies with larger populations are necessary to validate the possible value of this RRM1 SNP in gemcitabine-based chemotherapy.

## Conclusions

The RRM1 polymorphism -37C→A correlated with PFS in NSCLC patients treated with gemcitabine-based chemotherapy. No significant correlation was found between PBMC RRM1 mRNA expression and the efficacy of gemcitabine.

## Patients and Methods

### Patients

Advanced NSCLC patients treated at Guangdong General Hospital were enrolled. Eligibility criteria included a histological or cytological diagnosis of stage IIIB and IV NSCLC, WHO performance status (PS) of 0-1, age >18 years, no prior chemotherapy or thoracic radiation, and adequate bone marrow, liver, and kidney function. All patients were treated with gemcitabine/carboplatin regimen as a first line chemotherapy, patients received gemcitabine 1000 mg/m^2 ^on days 1 and 8, and carboplatin, AUC = 5, on day 1, every 21 days for a maximum of four cycles. Using the Response Evaluation Criteria in Solid Tumor Group (RECIST) guidelines, response was assessed with a computed tomography (CT) scan after two cycles of chemotherapy and was confirmed after four cycles. Patients have follow-up visit every 3 months with CT scan for 1 year. The study was approved by the Ethics Committee of the Guangdong General Hospital. Written informed consent was obtained from all patients.

### Sample collection

Before the first round of chemotherapy, a venous blood sample (4 mL) from each patient was collected in tubes containing EDTA (50 mmol/L). Total RNA was extracted from PBMCs using Trizol reagent (Invitrogen, Carlsbad, CA). Genomic DNA was extracted by the citrate sodium method, according to the protocol in the manual for Trizol LS reagent http://tools.invitrogen.com/content/sfs/manuals/10296010.pdf.

### RRM1 expression analysis

The cDNA was generated from RNA with a SuperScript™ III First-Strand Synthesis System (Invitrogen). Using an ABI PRISM 7000 Sequence Detection System (Applied Biosystems, Foster City, CA), real-time quantitative PCR for RRM1 and the housekeeping gene β-actin was conducted, with 5 ng of cDNA per reaction. The gene copy number of β-actin was used as an internal control. For standard curve determination, plasmids containing the same target sequences were used as standards; relative gene expression quantification was calculated according to the copy number of RRM1. The standardized copy number was determined by dividing the target copy number by the calibrator copy number.

### RRM1 SNP genotyping

To check for SNPs in RRM1 (-37C→A, 2455A→G, 2464G→A), PCR amplification of genomic DNA was performed, followed by direct sequencing. Primer pairs were designed based on the published RRM1 sequence (GenBank accession number AF107045): -37C→A primers, F-5'-TTAACCGCCTTTCCTCCG-3' and R-5'-GGGATTTGGATTGTTGCG-3'; 2455A→G and 2464G→A primers, F-5'-TTGGTGTGGAATGTCTAGTATTCTCAC-3' and R-5'-AAGTAGTTTGGCTACTGAAGACATGCT-3'. PCR reactions were performed in a total volume of 25 μL containing genomic DNA (25 ng), 1 μL of forward and reverse primers (10 μmol/L), 12.5 μL of PCR Master Mix (Tiangen Biotech, China), and ddH_2_O (8.5 μL). PCR cycling was performed with an initial denaturation at 94°C for 3 min, followed by 30 cycles of denaturation at 94°C for 30 s, annealing at 56°C for 30 s, and extension at 72°C for 30 s, with a final extension at 72°C for 5 min. PCR products were purified using a QIAquick Gel Extraction kit (Qiagen, Germany).

Direct sequencing of PCR products were performed with a 3100-Advant Genetic Analyzer (Applied Biosystems). The reaction mixture contained 1 μL of PCR products, 1.6 μL of forward and reverse primers (same as PCR primers), H_2_O (1.4 μL), and Bigdye (1 μL). The reaction mixture was denatured at 96°C for 1 min, followed by 25 cycles of 96°C for 10 s, 50°C for 5 s, and 60°C for 4 min. The Bigdye-labeled PCR products were sequenced using a Genetic Analyzer, and SNPs were checked by comparison with the published RRM1 sequence.

### Statistical analyses

Correlations between gene expression and the PS, gender, smoking status, age, histology, and other baseline characteristics were evaluated by logistic regression. Survival was calculated by Kaplan-Meier method, and the log-rank test was used to determine the level of significance between survival curves. The Kendall's tau correlation was used to determine correlations between SNPs and chemotherapy response or toxicity. Spearman correlation was used to test correlations between SNPs and gene expressions. Potential associations between gene expression levels and SNPs or response were compared with the Kruskal-Wallis test. All statistical calculations were performed with SPSS 13.0 (SPSS Inc., Chicago, IL). Two-sided *p*-values of less than 0.05 were deemed to indicate statistical significance.

## Competing interests

The authors declare that they have no competing interests.

## Authors' contributions

SD designed the study, carried out parts of these experiments and drafted the manuscript, ZC and ZX carried out the gene expression analysis. YH carried out the gene sequencing. ZW and XZ participated in the design of the study. YW and AG participated in its design and coordination and helped to draft the manuscript. HC and HY participated in the collection of samples and follow-up. All authors read and approved the final manuscript.
